# American Edition of Nothnagel’s Encyclopedia

**Published:** 1902-08

**Authors:** 


AMERICAN EDITION OF NOTHNAGEL'S ENCYCLOPEDIA.—DIPHTHERIA. By Wm. P. Northrup, M. D., of New York. MEASLES, SCARLET FEVER, AND GERMAN MEASLES. By Professor Dr. Th. von Jurgensen, Professor of Medicine in the University of Tubingen. Edited, with additions, by William P. Northrup, M. D., Professor of Pediatrics in the University and Bellevue Medical College, New York. Handsome octavo, 672 pages, illustrated, including 24 full-page plates, 3 of them in colors. Philadelphia and London: W. B. Saunders & Co. 1902. Cloth, $5.00 net; half Morocco, $6.00 net.

This volume, the third in the series of monographs issued under the editorial management of Prof. Nothnagel, is as excellent in every respect as are its predecessors. Prof. Jurgensen's article on "Measles" is by far the best and most complete contribution on the subject that has as yet appeared in the English language. It covers one hundred and eighty well illustrated pages, and contains, among other valuable records, a complete account of the famous "Faroe Island Epidemic." The subjects "Scarlatina" and "Rothelm" receive equally as full and thorough treatment at the hands of the same author.

Even more important than these to the average practitioner is the original and masterly article on "Diphtheria" by Dr. Northrup. We are not familiar with the German monograph for which this is substituted, but we are sure that it can in no way be superior. Beginning as Pathologist at the New York Foundling Hospital when the first bi-valve tubes were used, and having been associated with Dr. O'Dwyer while intubation tubes were being perfected, the author is certainly an authority on the surgical treatment of diphtheria.

The twenty-one half-tone figures illustrating the methods of performing intubation and the after methods of treatment are the best that it has ever been our pleasure to see. By a study of these the steps in the various operative procedures could, if necessary, be mastered without reference to the text.

Too much cannot be said in praise of the publisher for the mechanical make up of the work. The paper, printing, and binding are all that could possibly be desired. R.

